# Allopurinol Disrupts Purine Metabolism to Increase Damage in Experimental Colitis

**DOI:** 10.3390/cells13050373

**Published:** 2024-02-21

**Authors:** Corey S. Worledge, Rachael E. Kostelecky, Liheng Zhou, Geetha Bhagavatula, Sean P. Colgan, J. Scott Lee

**Affiliations:** 1 Mucosal Inflammation Program, Department of Medicine, University of Colorado Anschutz Medical Campus, Aurora, CO 80045, USA; corey.worledge@cuanschutz.edu (C.S.W.); rachael.kostelecky@cuanschutz.edu (R.E.K.); liheng.zhou@cuanschutz.edu (L.Z.); geetha.bhagavatula@childrenscolorado.org (G.B.); sean.colgan@cuanschutz.edu (S.P.C.); 2 Rocky Mountain Regional Veterans Affairs Medical Center, Aurora, CO 80045, USA

**Keywords:** allopurinol, purines, energy metabolism, wound healing, proliferation, inflammatory bowel disease, colitis, hypoxanthine

## Abstract

Inflammatory bowel disease (IBD) is marked by a state of chronic energy deficiency that limits gut tissue wound healing. This energy shortfall is partially due to microbiota dysbiosis, resulting in the loss of microbiota-derived metabolites, which the epithelium relies on for energy procurement. The role of microbiota-sourced purines, such as hypoxanthine, as substrates salvaged by the colonic epithelium for nucleotide biogenesis and energy balance, has recently been appreciated for homeostasis and wound healing. Allopurinol, a synthetic hypoxanthine isomer commonly prescribed to treat excess uric acid in the blood, inhibits the degradation of hypoxanthine by xanthine oxidase, but also inhibits purine salvage. Although the use of allopurinol is common, studies regarding how allopurinol influences the gastrointestinal tract during colitis are largely nonexistent. In this work, a series of *in vitro* and *in vivo* experiments were performed to dissect the relationship between allopurinol, allopurinol metabolites, and colonic epithelial metabolism and function in health and during disease. Of particular significance, the *in vivo* investigation identified that a therapeutically relevant allopurinol dose shifts adenylate and creatine metabolism, leading to AMPK dysregulation and disrupted proliferation to attenuate wound healing and increased tissue damage in murine experimental colitis. Collectively, these findings underscore the importance of purine salvage on cellular metabolism and gut health in the context of IBD and provide insight regarding the use of allopurinol in patients with IBD.

## 1. Introduction

Inflammatory bowel disease (IBD) is a chronic inflammatory condition of the gastrointestinal tract encompassing Crohn’s disease (CD) and ulcerative colitis (UC). A substantial component perpetuating the chronicity of these diseases is an energy deficit that limits gut tissue wound healing [1,2,3]. Much of this energy shortfall is due to dysbiosis shifting the composition and function of the microbiota and the resultant loss of microbiota-derived metabolites (MDMs), such as the short chain fatty acid butyrate, which the epithelium relies on as a primary fuel source [3,4]. A dysbiosis-induced loss of MDMs within the gut is detrimental to epithelial barrier function, and results in increased intestinal permeability of luminal antigens which stimulates an inappropriate host immune response and resultant inflammation [5,6]. This increase in intestinal permeability is a central component of the chronic disease state by allowing microbes and microbial components to enter the body, exacerbating local inflammation that promotes systemic, low-grade inflammation. This persistent inflammation may ultimately contribute to autoimmunity, in addition to the local inflammation feeding-back to perpetuate dysbiosis and the chronic disease state [7,8]. Continued disruption of the intestinal microbiota results in a profound loss of substrates for epithelial energy balance, and coincides with the overgrowth of pathogenic bacteria, leading to further energy depletion and loss of the homeostatic microenvironment [9,10].

Integral to the regulation of intestinal permeability, intestinal epithelial cells (IECs) line the surface of the intestine and establish a robust mucosal barrier. This barrier functions to facilitate the transit of critical nutrients, ions, and solutes either through cells or between them, and is composed of a network of cells interconnected through the protein complexes of the apical junctional complex (AJC) [8,11]. Constant cycling of proteins within the AJC is critical for proper barrier function, and the cytoskeletal function necessary for regulating the AJC can require as much as 20% of the total energy that IECs consume [12]. In addition, the mammalian epithelium is renewed every 4–5 days, making it one of the most proliferative tissues in the body, which also contributes to its high nucleotide requirement [13]. Accordingly, IECs are heavily reliant on purine metabolism, including both the salvage and *de novo* synthesis pathways, but preferentially salvage in the presence of exogenous purines because *de novo* synthesis is an energetically intensive metabolic process requiring eleven catalytic steps, five enzymes requiring adenosine triphosphate (ATP), and three amino acids to generate one molecule of inosine monophosphate (IMP) from phosphoribosyl pyrophosphate (PRPP) [14]. Conversely, salvage directly forms nucleotides from PRPP and Hpx, substantially alleviating the cellular energetic burden [15]. In addition to their intracellular roles, purines are also known to function as extracellular signaling molecules during active inflammation and support neutrophil–endothelial crosstalk necessary for the modulation of inflammation and vascular barrier function [16]. In epithelial tissues, Hpx was recently identified as a checkpoint metabolite in IECs that, when orally or microbially supplied, drives ATP genesis through the purine salvage pathway in support of cytoskeletal function, barrier function, and wound healing during experimental colitis [4,5].

Allopurinol is a synthetic hypoxanthine isomer commonly prescribed for the treatment of excess uric acid in the blood, and functions by preventing the degradation of hypoxanthine through competitive inhibition of xanthine oxidase (XO) [17]. XO, a primary metabolizer of hypoxanthine, is thought to directly bind and catabolize the purine nucleobase, producing first xanthine, and through subsequent catalysis, uric acid [18]. Allopurinol is not principally metabolized by xanthine oxidase *in vivo*, however, but by aldehyde oxidase, which yields the more active metabolite, oxipurinol [19]. Allopurinol and oxipurinol are both capable of ribosylation and yield several derivative compounds, including allopurinol riboside, a nucleoside analog of inosine with studied anti-parasite activity [20]. Additionally, an early pharmacological study on allopurinol pointed to a non-linear positive relationship between allopurinol riboside production and escalating doses of allopurinol [21]. This indicates potential metabolic interference by hypoxanthine-guanine phosphoribotransferase (HGPRT) or purine nucleoside phosphorylase (PNP), which have both been reported to produce the inosine analog, potentially due to the suppression or saturation of xanthine and aldehyde oxidase [21,22]. Although the use of allopurinol is common throughout the world, the impact that it and its metabolites have on the gastrointestinal tract (GI) is largely unstudied. Consequently, it is not known whether supplementation with allopurinol would be detrimental or provide the same protective effects during colitis as hypoxanthine. Outside of its use in the treatment of gout, allopurinol is given in combination with azathioprine to IBD patients who have no response to azathioprine alone, or who suffer more severe side effects from azathioprine [17]. 

To better understand how allopurinol and its metabolites influence colonic metabolism *in vitro* and *in vivo* we performed a series of experiments to assess the influence during active colitis and following recovery from colitis. Initial *in vitro* studies with allopurinol revealed that it impedes ATP production and energy distribution through influences on the creatine kinase circuit. Of particular significance, our studies identified a significant shift in adenylate flux, creatine, and colonic AMP-activated protein kinase (AMPK) that resulted in diminished IEC proliferation and increased tissue damage in colitis. Overall, these studies identify an allopurinol-associated loss of wound healing capacity in the intestine.

## 2. Materials and Methods

### 2.1. Cultured Cell Extraction 

T84 intestinal epithelial cells were grown and maintained as confluent monolayers in 1:1 Dulbecco’s modified Eagle’s medium (DMEM)–Ham’s F-12 medium with 10% FBS at 37 °C in 5% CO_2_ in room air [23]. For the electrophysiological measurements described below, T84 cells were plated on collagen-coated permeable supports as previously described in detail [23]. Monolayers were grown on 0.33 cm^2^ or 5.0 cm^2^ ring-supported polycarbonate filters (Costar, Cambridge MA) unless otherwise noted, and they were used 6–12 days after plating, as described previously [24]. Extractions were performed on ice, with centrifugations performed at 4 °C. Transwell permeable support inserts (Costar; 5 cm^2^, 0.4 μM) containing T84 cells were put on ice and washed five times in PBS. The insert membrane was subsequently removed and submerged in a 1.5 mL microcentrifuge tube containing 500 μL of 80% MeOH. The microcentrifuge tube containing the sample was then placed in liquid N_2_ until completely frozen, removed from the nitrogen, thawed on ice, and vortexed for 10 s. The sample was centrifuged for 10 min at 18,000× *g*, and the supernatant was then transferred to a new Eppendorf tube (Fisher Scientific, Waltham USA). Another 500 μL of 80% MeOH was added to the sample, and the same extraction process was repeated two more times. The resulting 1.5 mL of extract in methanol was subjected to vacuum centrifuge (Eppendorf Vacufuge, Hamburg Germany) at room temperature for ~four hours. The dried extract was dissolved in 500 μL of HPLC “mobile phase A” (details included below) and then filtered (Whatman puradisc 4, 0.45 μM, nylon, Cytiva Life Sciences, Marlborough, MA, USA) into vials for HPLC injection.

### 2.2. Tissue Extraction 

A total of 10–30 mg of whole, distal colon was placed in pre-weighed 1.5 mL microcentrifuge tubes, weighed again to account for colon weight, then quickly placed in 500 μL of 80% MeOH and flash-frozen in liquid N_2_ to halt metabolism. The frozen tissue was then stored at −80 °C before extraction was carried out. The extraction procedure was identical to the one described for cultured cells above, adding an additional sonication step for five seconds prior to each of the three extractions (BioLogics Inc., Manassas, VA, USA, 150 V/T Ultrasonic Homogenizer, power output ~20). Briefly, the tissue was first sonicated for 3 × 10 s (BioLogics Inc., 150 V/T Ultrasonic Homogenizer, power output ~20). The sample was then placed in liquid nitrogen until frozen, removed and thawed, and vortexed for ~10 s. The sample was centrifuged for 10 min at 18,000× *g* and 4 °C, and the supernatant transferred to a new Eppendorf tube. Another 500 μL of 80% MeOH was then added to the sample, the tissue was resuspended, and the extraction process was repeated twice more. The resulting 1.5 mL of extract was dried via an Eppendorf vacufuge at room temperature. The dried extract was dissolved in 300 μL of mq water and filtered (Whatman Puradisc 4, 0.45 μm, nylon) into vials for HPLC injection.

### 2.3. Extracellular Fecal Extraction

Fecal pellets were removed from −80 °C conditions and dispersed in 400 µL of ice-cold Milli-Q (mq) water. All extractions were performed on ice. The fecal pellet was manually dispersed using a 1 mL pipette tip and then centrifuged at 2000× *g* and 4 °C for 10 min. The supernatant was then transferred to a new tube, another 400 µL of mq water added to the fecal matter, and then the matter resuspended. The extraction process was then repeated two more times, producing a total of 1.2 mL of extract. The extract was then filtered through a Vivaspin 5000 MWCO PES column (Sartorius Stedim Biotech, Gottingen, Germany) and submitted to metabolite analyses.

### 2.4. HPLC Analysis 

Analyses were performed on an Agilent Technologies 1260 Infinity HPLC (Santa Clara, CA, USA) using a Phenomenex Luna 5 μm C18 column (Torrance, CA, USA; mobile phase A = 50 mM KH_2_PO_4_, 5 mM tetrabutylammonium bisulfate, pH 6.25; mobile phase B, acetonitrile, column temperature, 30 °C; flow rate, 1 mL/min). Chromatographic separation of the metabolites was performed using a combination of isocratic and gradient methods, including column washing and equilibration periods at the end (0 min, 100% A; 7 min, 100% A; 10 min, 97% A; 18 min, 97% A; 45 min, 85% A; 60 min, 50% A; 80 min, 50% A; 90 min, 100% A; 135 min, 100% A). Metabolites were detected by absorption at wavelengths of 210, 254, and 280 nM. Absorbance spectra and retention times were verified by co-injection with measured standards.

### 2.5. HPLC-ESI MS Analysis

Metabolites were analyzed as previously described with minor variations [25]. Analyses were performed on an Agilent Technologies 1260 Infinity II LC/MSD iQ (Santa Clara, CA, USA) with electrospray ionization (ESI) mass detection. Negative ion mass-to-charge ratios (*m*/*z*) were scanned from 50 to 500. The metabolite extracts were chromatographed using a Sepax Carbomix column (Newark, DE, USA; Pb-Np5:8%, 5 μm, non-porous, 4.6 × 300 mm) (mobile phase A: water; mobile phase B: acetonitrile; column temperature, 75 °C). Chromatographic separation was performed using a combination of isocratic and gradient methods, including column washing and equilibration periods at the end (0 min: 100% A, 0.12 mL/min; 60 min: 100% A, 0.12 mL/min; 70 min: 70% A, 0.5 mL/min; 145 min: 70% A, 0.5 mL/min; 150 min: 100% A, 0.5 mL/min; 164 min, 100% A, 0.5 mL/min; 165 min, 100% A, 0.12 mL/min; 170 min, 100% A, 0.12 mL/min). The metabolites were detected by the masses of their negatively charged ions (M-1 ± 0.05; 145 *m*/*z*; succinate, 115 *m*/*z*; butyrate, 87 *m*/*z*; propionate, 73 *m*/*z*; acetate, 59 *m*/*z*; lactate, 89 *m*/*z*; fructose/glucose-6-phosphate, 258 *m*/*z*; ribose-5-phosphate, 229 *m*/*z*), with their retention times and m/z verified by co-injection with authentic standards.

### 2.6. Histological and Immunofluorescent Analyses 

Paraffin embedding of tissue and slide preparation (blank and H&E) was performed by the University of Colorado AMC Department of Pathology histology laboratory. Slides were deparaffinized through a series of washes: xylene, 2 × 3 min; xylene:ethanol::1:1, 3 min; ethanol, 2 × 3 min; 95% ethanol, 3 min; 70% ethanol, 3 min; 50% ethanol, 3 min; rinse in cold tap water.

H&E Scoring: Histological examination was performed on samples of the distal colon. All histological quantitation was performed in a blinded fashion, using a previously described scoring system [26]. The three independent parameters measured were severity of inflammation (0–3: none, slight, moderate, severe), extent of injury (0–3: none, mucosal, mucosal and submucosal, transmural), and crypt damage (0–4: none, basal 1/3 damaged, basal 2/3 damaged, only surface epithelium intact, entire crypt and epithelium lost). The score of each parameter was multiplied by a factor reflecting the percentage of tissue involvement (x1: 0–25%, x2: 26–50%, x3: 51–75%, x4: 76–100%) and all numbers were summed for a maximum possible score of 40.

Immunofluorescence: Deparaffinized slides were placed in Tris-EDTA buffer (10 mM Tris base, 1 mM EDTA, 0.05% Tween 20, pH 9.0) for heat-induced epitope retrieval (HIER) (Biocare Medical Decloaking Chamber, DC2008US). HIER was performed at 80 °C for 1 h (Ki67, AMPK-P) and the slides then equilibrated to room temperature. The slides were washed 2 × 5 min in TBS wash buffer (50 mM Tris, 150 mM NaCl, 0.025% Triton X-100, pH 7.6), the tissue was permeabilized by immersion in TBS wash buffer containing 0.2% Triton X-100 for 8 min, and then the slides were washed again 2 × 5 min in TBS wash buffer. The tissue was blocked using 10% normal goat serum and 1% bovine serum albumin (BSA) in Tris-buffered saline (TBS, 50 mM Tris, 150 mM NaCL, pH 7.6) for 2 h at room temperature. Primary antibody in TBS containing 1% BSA (Ki67, 1:100; AMPK-P, 1:50) was then added to the tissue, and the slides were incubated overnight at 4 °C. The slides were then washed 2 × 5 min in TBS wash buffer and secondary antibody was added (1:500 in 1% BSA TBS) and incubated for 1 h at room temperature. Then, the slides were rinsed for 3 × 5 min in TBS, a coverslip added using ProLong Diamond Mountant with DAPI, and the slides were cured for 24 h. Fluorescent images of the tissue were taken using a Zeiss AxioCam MRc 5 (Oberkochen, Baden-Wurttemberg, Germany) at 100× or 200× magnification. Multiple, nonoverlapping images of each tissue section from each mouse group (n = 5) were taken, and all images were used for quantification. Images were processed and quantitated using the Zeiss Zen2 program. Immunofluorescence was quantified by drawing regions of interest around the epithelium and was normalized to DAPI (target/DAPI, fluorescence sum/sum).

### 2.7. Vertebrate Animal Use 

The University of Colorado Anschutz Medical Campus (AMC) animal management program is accredited by the American Association for the Accreditation of Laboratory Animal Care (AAALAC) and meets the National Institutes of Health standards as set forth in the Guide for the Care and Use of Laboratory Animals (DHHS Publication No. (NIH) 85–23). The institution also accepts as mandatory the PHS Policy on Human Care and Use of Laboratory Animals by Awardee Institutions and the NIH Principles for the Utilization and Care of Vertebrate Animals Used in Testing, Research, and Training. 

Eight-to-twelve week-old male and female C57BL/6 mice whose lineage came from the Jackson Laboratories were used. Both species- and sex-related differences have been reported in aldehyde oxidase activity, and sex-related differences in serum uric acid (UA) have been noted in humans [27,28,29]. After noting wide variability among the male subjects, and between the male and female subjects, further studies were limited to female mice. Mice in the treatment group (n = 10) were administered allopurinol through drinking water supplementation at a therapeutically relevant dose (21.875 mg allopurinol in 350 mL H_2_O for an estimated 10 mg/kg dose per day) based on the studies used in determining U.S. pediatric dosing guidelines [30,31,32], while those in the control group (n = 9) were administered drinking water free of allopurinol. Allopurinol was solubilized in the drinking water by heating and remained soluble after cooling. All animal work was approved by the Institutional Animal Care and Use Committee (IACUC) at the University of Colorado (protocol code 00182, approved 23 May 2021).

Disease Activity Index (DAI) scoring criteria: Combined score of weight loss, stool consistency, and bleeding, divided by 3. A score of 0 for weight loss indicates no weight loss, 1 indicates 1–5% weight loss, 2 indicates 6–10%, 3 indicates 11–15%, and 4 indicates loss of greater than 15% body weight. A score of 0 for stool consistency indicates normal stool consistency, a score of 2 indicates loose stool consistency, and a score of 4 indicates diarrhea. A score of 0 for rectal bleeding indicates no rectal bleeding, a score of 2 indicates gross bleeding, a score of 3 indicates gross bleeding for more than one day, and a score of 4 indicates gross bleeding for greater than two days. Normal stool is defined as well formed pellets; loose stool is defined as pasty stool that does not stick to the anus; diarrhea is defined as liquid stool that sticks to the anus [33].

### 2.8. Seahorse XF Assay

T84 cells were grown in a 96-well XF cell culture plate (Agilent Technologies, Santa Clara, CA, USA) using the previously described method for culturing cells for 24 h with no treatment, 10 μM, 100 μM, or 1 mM allopurinol added to the medium. After 24 h, the medium was replaced with Seahorse XF DMEM (Agilent Technologies) with added glucose, pyruvate, and L-glutamine, and placed in a non-CO_2_ incubator for one h. The medium was then replaced again with Seahorse XF DMEM, incubated for 20 min, and then taken to the University of Colorado Nutrition Obesity Research Center (NORC) for analysis via the real-time ATP rate assay. After analysis, normalization was undertaken by cell count.

### 2.9. Statistical Analyses

*In vitro* data are expressed as mean ± S.D., and in vivo data are shown as mean ± S.E. Statistical analyses were performed in GraphPad Prism 9 using an unpaired two-tailed parametric *t*-test with Welch’s correction for direct comparisons. Statistical differences were considered significant when *p* ≤ 0.05. Additional statistical details can be found in the figure legends.

## 3. Results

### 3.1. Allopurinol Modulates Intestinal Epithelial Cell Energy Metabolism

**Figure 1:**
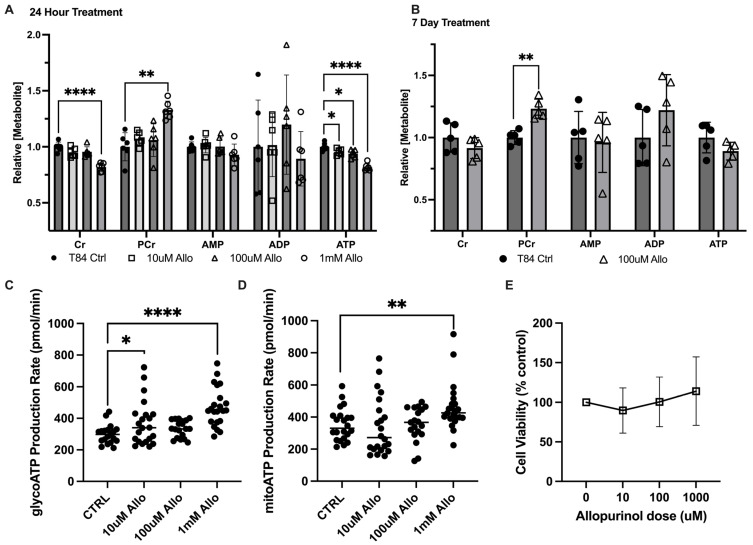
Metabolic responses of intestinal epithelial cells to allopurinol. (**A**) Relative energy metabolite response to a changing dose of allopurinol as measured by HPLC; n = 6, t = 24 h. (**B**) Relative energy metabolite response to chronic treatment with allopurinol treated cells as measured by HPLC; n = 5, t = 7 days. (**C**) Glycolytic ATP production as measured by Seahorse XF; n = 24, t = 24 h pre-treatment with allopurinol. (**D**) Oxidative phosphorylative ATP production as measured by Seahorse XF; n = 24, t = 24 h pre-treatment with allopurinol. (**E**) Cell viability as measured by Cyquant MTT Viability Assay; n = 24, t = 24 h pre-treatment with allopurinol. Data are presented as mean ± S.D.; *, *p* < 0.05; **, *p* < 0.001; ****, *p* < 0.0001. Cr, creatine; PCr, phosphocreatine; AMP, adenosine monophosphate; ADP, adenosine diphosphate; ATP, adenosine triphosphate.

To assess the influence of allopurinol on energy metabolism, model T84 intestinal epithelial cells (IECs) were treated with 10 μM, 100 μM, or 1 mM allopurinol for 24 h, and cellular extracts were analyzed by high-performance liquid chromatography (HPLC). Similar to previous work with an overnight 1 mM allopurinol dose, analyses from all three doses revealed no significant difference in intracellular adenosine monophosphate (AMP) or adenosine diphosphate (ADP), with the 1 mM dose increasing phosphocreatine 24.7% (*p* = 0.0005) relative to the control (Figure 1A) [5]. By contrast, it was observed that allopurinol appeared to elicit an inverse, dose-dependent response on intracellular adenosine triphosphate (ATP) concentrations. Notably, the 10 μM dose decreased ATP by 4.5% (*p* = 0.03), while the 100 μM dose decreased ATP by 6.1% (*p* = 0.01), and at the 1 mM dose allopurinol was found to decrease ATP levels by 18.2% (*p* < 0.00005). Additionally, the 1 mM treatment induced an 18% intracellular creatine decrease (*p* = 0.000014). Further analysis after a longer, seven-day allopurinol treatment (media and allopurinol refreshed daily) yielded similar results, revealing 18.8% increased phosphocreatine (*p* < 0.005), a consistent but insignificant 8.3% decrease in creatine (ns), and a 12.2% decrease in ATP (ns) (Figure 1B).

Given the central role of ATP in energy metabolism, analyses were extended to include the Seahorse XF real-time ATP rate assay (Agilent). In T84 cells pre-treated with 10 μM, 100 μM, or 1 mM allopurinol, glycolytic ATP production was found to increase by 19% (*p* = 0.0416), 10.4% (ns), and 36.4% (*p* < 0.0001), respectively, over that of controls (Figure 1C). In addition, mitochondrial ATP production was found to be increased by 23.7% (*p* = 0.0069) in 1 mM allopurinol-treated T84 cells (Figure 1D). To rule out cell death due to allopurinol toxicity as a mechanism for loss of ATP and creatine, an MTT viability assay was performed on confluent T84 cells treated with 10 μM, 100 μM, or 1 mM allopurinol for 24 h. No significant difference from controls was noted at any dose; however, we did note a trend toward higher viability with greater allopurinol dose (Figure 1E), demonstrating that allopurinol treatment was not causing increased cell death over that of untreated controls. Together, these results indicate that allopurinol is capable of modulating *in vitro* epithelial energy metabolism with a PCr increase appearing as a consistent biomarker. The increase in production of ATP, taken with decreased intracellular ATP and increased PCr, additionally demonstrates the significance of purine salvage to nucleotide genesis and energy balance, and suggests the addition of allopurinol might induce cellular stress through inhibition of salvage and costly activation of *de novo* purine biosynthesis to offset the loss of salvage products.

### 3.2. Allopurinol Riboside Diminishes Cellular and Extracellular Creatine Concentrations

**Figure 2:**
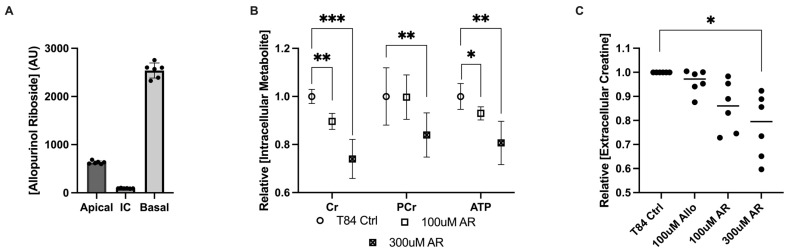
Metabolite responses of intestinal epithelial cells to allopurinol riboside. (**A**) Intra- and extracellular allopurinol riboside concentrations measured 24 h after treatment with allopurinol. (**B**) Metabolite responses for allopurinol riboside (100 μM and 300 μM)-treated T84 cells; n = 6, t = 24 h. (**C**) Metabolite response between intracellular allopurinol/allopurinol riboside and extracellular creatine concentrations as measured by HPLC; n = 6, t = 24 h. Data are presented as mean ± S.D.; *, *p* < 0.05; **, *p* < 0.01; ***, *p* < 0.001. Cr, creatine; PCr, phosphocreatine; ATP, adenosine triphosphate; AR, allopurinol riboside.

In addition to increased phosphocreatine, it was noted that treatment with 100 μM and 1 mM allopurinol generated a metabolite consistent with allopurinol riboside (AR), as was previously reported [5]. This AR appeared to be primarily excreted basolaterally but was also present both apically and intracellularly (Figure 2A). As AR is an allopurinol metabolite that may influence purine metabolism, AR was investigated independent of allopurinol in T84 model cells. Analogous to allopurinol, treatment with AR for 24 h induced a dose-dependent decrease in intracellular Cr and ATP concentrations, leading to a 10.4% decrease in Cr (*p* = 0.0003) and a 7% decrease in ATP (*p* = 0.02) with 100 μM AR, and a 26% decrease in Cr (*p* < 0.0005), which correlated with 19.3% decreased ATP at the 300 μM dose. Interestingly, no difference in PCr concentration was observed at the 100 μM dose, but PCr was decreased by 16% at 300 μM (*p* = 0.03), demonstrating that the effect of AR on creatine metabolism differed from that of allopurinol (Figure 2B). To assess whether the decreased Cr levels observed with allopurinol and the riboside derivative could be related to transport, the extracellular medium was analyzed. Treatment with allopurinol was not found to significantly change extracellular creatine concentrations from that of controls; however, AR treatment coincided with a 20.6% decrease in extracellular creatine (*p* = 0.03) and a corresponding but nonsignificant 11.2% decrease in phosphocreatine at the 100 μM dose. When administered at a higher 300 μM dose, Cr was observed to decrease by 22.3% (*p* = 0.007) (Figure 2C), suggesting that AR was not influencing the extracellular transport of Cr. Interestingly, while in allopurinol-treated cells diminished Cr was associated with a corresponding increase in PCr, Cr loss in AR-treated cells could not be accounted for, either as PCr or creatinine, suggesting that AR might influence the synthesis or degradation of creatine.

### 3.3. Allopurinol Sensitizes the Epithelium to Metabolic Insult in Acute Colitis

The salvage of hypoxanthine for ATP biosynthesis is a critical process in the body that supports epithelial barrier function and wound healing [28]. To define the influence of allopurinol *in vivo*, C57BL/6 mice were given either a therapeutically relevant allopurinol dose in drinking water, or in the case of controls, drinking water which did not contain allopurinol. The introduction of allopurinol into drinking water did not influence animal weight, activity, or volume of water consumed (Figure S3A). After seven days of allopurinol supplementation, chemical colitis was induced in both groups with a five-day course of 3% dextran sodium sulfate (DSS). Mice were sacrificed 2 days following cessation of DSS (peak disease) or were allowed to heal for 7 days (resolution phase). Physically, there were no notable differences in weight-loss, bleeding, or diarrhea (components of the Disease Activity Index widely used to assess disease severity in DSS colitis) with allopurinol supplementation compared to controls (Figure S3B–E).

**Figure 3:**
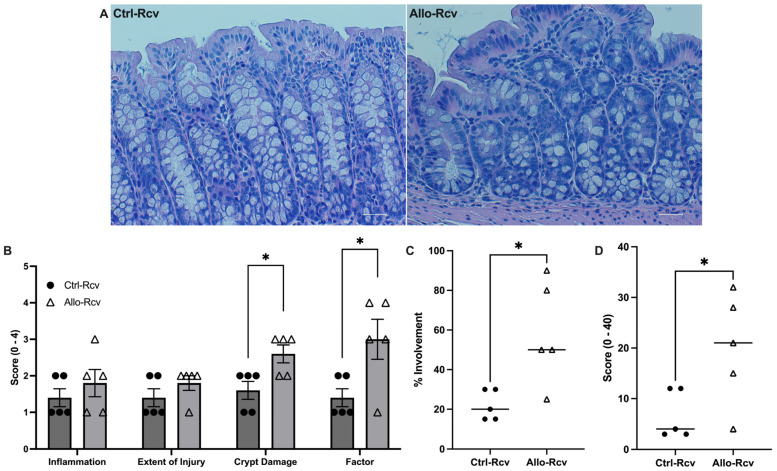
Murine histological response to allopurinol supplementation following experimental colitis. (**A**) Representative hematoxylin and eosin (H&E) staining images of control (Ctrl-Rcv) and allopurinol-treated (Allo-Rcv) mice following recovery from DSS colitis. Scale bars, 50 μm. (**B**) Histological scoring of colitis symptoms. Three parameters measured were severity of inflammation (0–3: none, slight, moderate, severe), extent of injury (0–3: none, mucosal, mucosal and submucosal, transmural), and crypt damage (0–4: none, basal 1/3 damaged, basal 2/3 damaged, only surface epithelium intact, entire crypt and epithelium lost). The score of each parameter was multiplied by a factor reflecting the percentage of tissue involved (x1: 0–25%, x2: 26–50%, x3: 51–75%, x4: 76–100%). (**C**) Percentage of tissue affected in control (n = 5) and allopurinol-treated (n = 5) mice following colitis. (**D**) Total histological score of control and allopurinol-treated mice following colitis calculated by multiplying the score of each parameter by a factor reflecting the percentage of tissue involvement; all numbers were summed for a maximum possible score of 40. Data are presented as mean ± S.E. (error bars). *, *p* < 0.05.

Analysis of hematoxylin and eosin (H&E)-stained colonic tissue collected during peak colitis (two days following removal of DSS) showed no significant difference between groups; however, tissue from colitis resolution revealed 38% increased crypt damage among mice treated with allopurinol (*p* < 0.05), as well as 62% increased overall tissue involvement (Figure S4 and Figure 3A), which suggested the tissue damage had extended further into the submucosa than controls (*p* < 0.05). This analysis yielded a 66% increase in the overall scoring of colitis over controls (*p* < 0.05), reinforcing the importance of purine salvage for epithelial health (Figure 3B–D). Taken together, these findings underscore an allopurinol-induced inability to properly recover from the DSS colitic insult.

### 3.4. Allopurinol Inhibits Epithelial Proliferation and Downregulates Activated AMPK during Recovery

**Figure 4:**
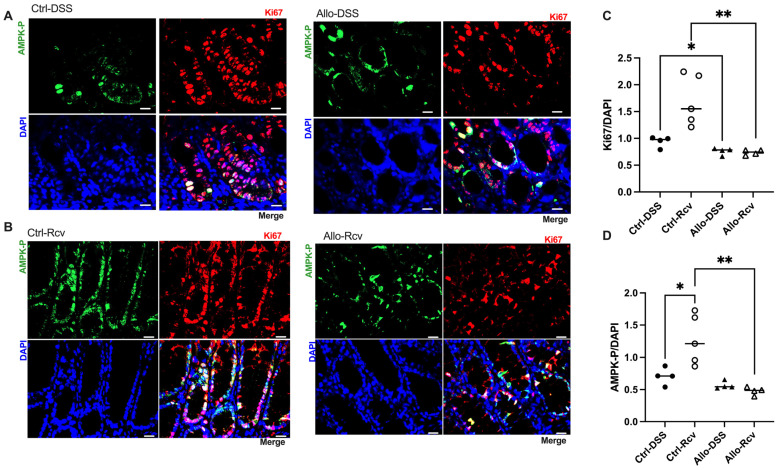
Effects of allopurinol on proliferation and wound healing in experimental colitis. (**A**) Representative images of control and allopurinol-treated mouse cohort immunofluorescent analyses at peak colitis. Scale bars, 20 μm. (**B**) Representative images of control and allopurinol-treated mouse cohort immunofluorescent analyses in recovery from colitis. Scale bars, 20 μm. (**C**) Murine colonic Ki67 immunofluorescent analyses of control and allopurinol-supplemented mice during peak colitis and following recovery from colitis. (**D**) Murine colonic activated AMPK (AMPK-P) immunofluorescent analyses of control and allopurinol-supplemented mice during peak colitis and following recovery from colitis. Immunofluorescent data points represent an average of 10 quantitated images (n = 4–5 mice). Data are presented as mean ± SEM (error bars). *, *p* < 0.05; **, *p* < 0.01).

Because of the importance of purine salvage in energy balance and proliferation [4], colon tissue analyses were extended to AMPK and Ki67 as markers. Immunofluorescence analysis of DSS-treated tissue revealed no change in active AMPK (AMPK-P), but an 18.9% decrease in Ki67 (*p* = 0.03) in allopurinol treated tissues at peak disease was noted, suggesting epithelial metabolic dysregulation (Figure 4A,C). This was further supported by immunofluorescence of recovered tissue for AMPK and Ki67, which revealed stark differences between the control and allopurinol-treated groups, with AMPK-P decreasing by 62.5% compared to control mice (*p* < 0.01), and a concurrent 56.8% decrease in Ki67 expression (*p* < 0.01) (Figure 4A–D). Given that the expression of Ki67 should be elevated as a marker of increased proliferation following cellular damage, these results were taken to reflect a vastly disturbed colonic metabolic microenvironment. Likewise, AMPK-P is elevated in response to energy depletion, and, as such, should be appropriately elevated to support energetically taxing wound healing [34,35]. Taken with a recent study, which used a transgenic AMPK KO mouse model to show delayed epithelial repair during DSS colitis and attributed the changes to decreased IEC proliferation, epithelial barrier maturation, and differentiation during the recovery phase of DSS colitis, these data further support our observations [36]. Indeed, through comparison of immunofluorescence at peak colitis and during recovery, it was observed that the control group showed slightly increased AMPK-P:DAPI and Ki67:DAPI at peak disease but displayed an increase in expression during recovery, while colon tissue in mice receiving allopurinol showed no initiation of these wound healing responses (Figure 4C,D).

### 3.5. Disruption of Purine Salvage by Allopurinol Induces Fecal and Colonic Tissue Metabolic Changes

**Figure 5:**
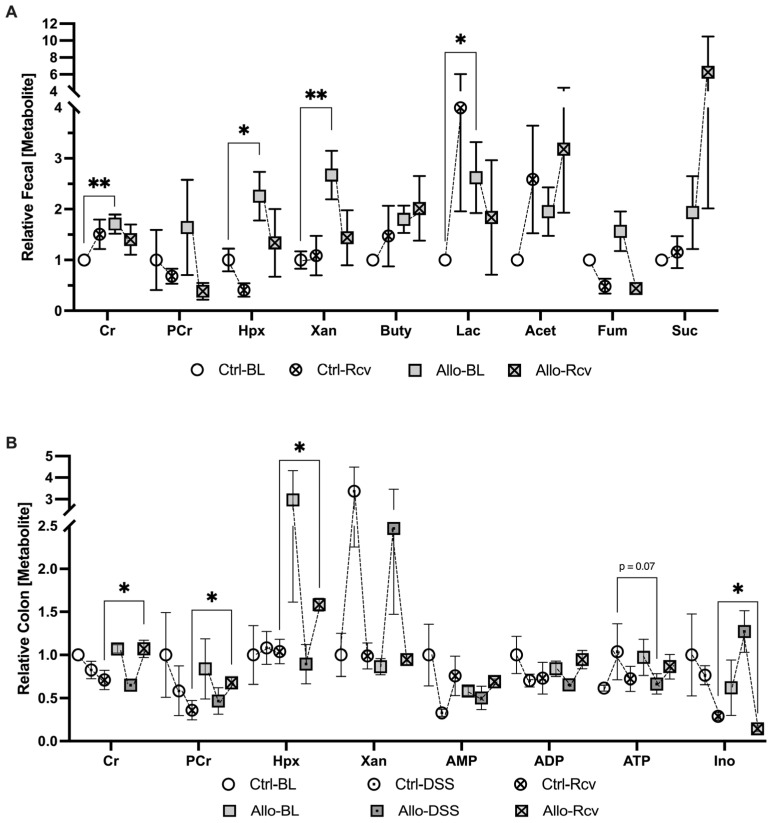
Metabolic analysis of the impact of allopurinol treatment before, during, and after colitis. (**A**) Extracellular metabolites in murine fecal extracts from baseline control (Ctrl-BL), colitis-recovered control (Ctrl-Rcv), baseline allopurinol-treated (Allo-BL) and colitis-recovered allopurinol-treated mice (Allo-Rcv; n = 5–10). (**B**) Colon tissue adenylate and energetic metabolite analyses from baseline control (Ctrl-BL), peak colitis control (Ctrl-DSS), colitis-recovered control (Ctrl-Rcv), baseline allopurinol-treated (Allo-BL), peak colitis allopurinol-treated (Allo-DSS and colitis-recovered allopurinol-treated mice (Allo-Rcv; n = 4–5). Data are represented as mean ± SEM. *, *p* < 0.05, **, *p* < 0.01; Cr, creatine; PCr, phosphocreatine; Hpx, hypoxanthine; Xan, xanthine; AMP, adenosine monophosphate; ADP, adenosine diphosphate; ATP, adenosine triphosphate; Ino, inosine; Buty, butyrate; Acet, Acetate; Fum, fumarate; Suc, succinate.

Despite the minimal physical change noted at baseline, extracellular fecal metabolite analyses revealed a striking 63% increase in lactate (*p* = 0.039), indicating either increased lactate production and/or decreased consumption by colonic microbiota, shifted tissue metabolism towards glycolysis, or a combination of the three, and suggests microbiota dysbiosis in all instances (Figure 5A) [37,38]. Additionally, fecal creatine was found to be elevated by a sizeable 46% (*p* = 0.004), demonstrating that allopurinol was capable of modulating creatine concentrations in a living system. It was not surprising to see an increase in available fecal purine, given that allopurinol inhibits the degradation and salvage of hypoxanthine and xanthine; however, the magnitude was unexpected. Fecal xanthine was found to increase by 57% (*p* = 0.01), while hypoxanthine levels increased by 55.6% (*p* = 0.035) compared to controls. Fecal samples at peak disease were not collected due to a lack of pellet formation. Conversely, recovered fecal metabolic analyses revealed no significant difference in fecal lactate, creatine, xanthine, or hypoxanthine, despite the earlier increases.

Baseline host colon tissue analyses revealed no significant difference in the levels of lactate, creatine, or xanthine, further supporting the idea that related elevated fecal analytes were the result of disruptions to the microbiota, secretion by the epithelium, or were the result of a profound loss in the ability of the epithelium to uptake nutrients from the lumen. Taken together with baseline fecal analytes, these initial results confirm that allopurinol affects creatine metabolism *in vivo*, though the observed excess luminal creatine did not appear to arise from losses in colonic tissue, and there was no significant change in measurable adenylate flux despite a nearly 3-fold increase in tissue Hpx. At peak disease however, a potential metabolic state of energy deficiency manifested as 49.5% decreased tissue ATP (*p* = 0.07) (Figure 5B) suggesting allopurinol was perturbing energy balance via purine salvage inhibition, which tracked with the significant decrease in tissue Ki67 in allopurinol treated tissues during this early state of wound healing. This effect was also manifested in recovered tissue analytes which indicated a 33% increase in epithelial creatine among the allopurinol-treated group, while phosphocreatine levels corresponded to those from *in vitro* study and increased by 46% over controls (*p* < 0.05). Taken with our earlier results, the simultaneous 33.4% increase in Hpx (*p* < 0.05), and stable adenylate nucleotide concentrations demonstrated that Hpx salvage inhibition was still disrupting energy balance. Collectively, these data suggest allopurinol administration prior to a colitis event triggers a more severe disease state by attenuating the adenylate flux needed to repair the intestinal epithelium following a metabolic insult, and significantly attenuates wound healing by downregulating epithelial proliferation and AMPK signaling,

## 4. Discussion

Purines form two of the four building blocks for DNA and RNA, namely the energetic molecules ATP and GTP, and are required to sustain all life. Accordingly, generation of purines is governed by two pathways that work in combination to maintain energy balance; namely the *de novo* purine biosynthesis pathway and the salvage pathway. *De novo* synthesis is an energetically intensive metabolic process requiring eleven catalytic steps, five enzymes requiring ATP, and three amino acids to generate one molecule of IMP from PRPP [39]. Conversely, salvage directly forms IMP from PRPP and Hpx, leaving energy available to fuel other cellular processes, such as nucleic acid synthesis [40]. Mammalian cells prefer salvage over *de novo* synthesis, which is regulated through the allosteric inhibition of amidophosphoribosyltransferase (ATase) by salvage products. This ensures that energy is not expended on *de novo* synthesis until salvage substrates are depleted [14]. In the present work, administration of allopurinol *in vitro* or in a murine model to inhibit adenylate flux generally manifested as accumulated phosphocreatine and hypoxanthine and depleted ATP and Cr. We presume that the observed accumulation of hypoxanthine *in vivo* indicates successful inhibition of XO, but that it also signals energetic stress. Under normal physiological conditions, tissue hypoxanthine levels are quite low; however, microbiota-sourced hypoxanthine is abundant in the lumen and can be taken up by the colonic epithelium as needed and used to generate ATP from IMP. After IMP is subsequently converted to AMP, adenylate kinase then drives salvage by catalyzing the transfer of a phosphate from ATP to convert the newly biosynthesized AMP, generating two molecules of ADP. At this point the ADP can then be converted to ATP via creatine kinase and PCr, glycolysis, and/or ATP synthase to complete purine salvage. We postulate that the inhibition of purine salvage by allopurinol incurs substantial energy redirection from distribution for cellular processes towards *de novo* synthesis, with this energy sequestration underscored by purinosome localization to the mitochondria [41,42]. As energy distribution for cellular processes is substantially mediated by creatine kinases (CKs), PCr appears to function as a purine generation-related biomarker. When purine salvage is disrupted due to inhibition or a lack of exogenous purine supply, the energy redirected to energetically intensive *de novo* purine biosynthesis and resultant stagnation in distribution appears to manifest as a PCr increase [4]. This is supported by the data from our *in vitro* ATP rate assay, which revealed increased glycolytic and mitochondrial ATP regeneration in purine salvage-inhibited cells that manifested as decreased ATP and increased PCr.

Elevations in fecal lactate have long been associated with IBD, and a recent study showed that lactate-utilizing bacteria (LUB) ameliorate experimental murine colitis, reinforcing the notion that colitis is associated with dysbiotic metabolism [43,44]. It is interesting to note that we observed ~2-fold allopurinol-induced increase in murine fecal lactate, Cr, and Xan, with a notable but insignificant Hpx increase, given the number of redundant and compensatory systems that biological organisms utilize to guard metabolic pathways against environmental perturbations [45,46,47]. This shift in creatine and purine mirrors changes observed in experimental models of IBD [2,48]. As these metabolites are regularly transported by microbes and the host, it is difficult to determine their source without further experimentation, but the lack of associating tissue shifts other than a similar insignificant Hpx increase suggests microbiota dysbiosis as a significant component. Altogether, the metabolic disruptions incurred by allopurinol at baseline primarily manifested in steady-state fecal metabolite levels and may signal an inability of the host tissue to utilize microbiota-sourced purine nucleobases. It is intriguing to consider a similar inhibition of purine nucleobase utilization and/or production in the microbiota.

When subjected to DSS colitis, disease activity index scoring that assesses weight loss, bleeding, and diarrhea revealed that allopurinol treatment did not substantially influence colitis pathogenesis (Figure S3). Despite the minimal difference, histology provided valuable visual data on the health of the epithelium. H&E scoring, while not significant, revealed an increase in colitis markers in four of the five treatment mice which was largely attributable to the sheer extent of tissue injury, and in some cases had resulted in near-complete crypt destruction. Despite a lack in physiological differences, a halving in tissue ATP (*p* = 0.07) signaled allopurinol-induced energetic stress (Figure 5B). In addition, immunofluorescent analyses for AMPK and Ki67 activity revealed significantly attenuated proliferation in allopurinol-treated tissues which we attributed to inhibited purine salvage decreasing nucleotide biogenesis and cellular energy. This significant decrease in colonic Ki67, taken with the depleted tissue ATP that was observed, highlights the risk of inhibiting purine salvage in highly proliferative tissues (e.g., epithelial surfaces), especially after stress.

It is largely accepted that recurrent injury to and incomplete healing of the intestinal epithelium predisposes the immune system for reactivation in IBD [49]. Given our observations during colitis, it seemed likely that allopurinol would also attenuate mucosal wound healing, aligning with our emerging concept that allopurinol was sensitizing the colonic epithelium to more severe colitis as a result of metabolic epithelial starvation. In our situation, epithelial starvation was observable by the inability of the epithelium to utilize microbiota-sourced nutrients. This concept is supported by the work of Roediger, who first proposed the “starved gut hypothesis” in IBD, suggesting that nutritional colitis occurred when colonic epithelial cells were unable to utilize SCFAs, despite their abundant presence [50]. It became evident after the recovery period that allopurinol incurred an analogous starvation that impaired wound healing. Histological analyses revealed significantly increased crypt damage, factor, and tissue involvement in the treatment group, which very likely resulted from the inability to capitalize on present metabolic substrates. It was interesting to note that inosine was found to be elevated during colitis while Hpx was decreased, and was significantly decreased following colitis, when Hpx was significantly increased. This observation implies that allopurinol and its metabolites may play different roles in the body during different physiological states and suggests some capacity of PNP to properly function in the presence of allopurinol during colitis. This effect may be due to an increase in xanthine oxidase expression during inflammation, which could sequester the drug, given that XO has the greater binding affinity for allopurinol, increasing PNP availability for catalysis [17]. Additionally, some studies have suggested that allopurinol may increase extracellular adenosine in vitro, which is rapidly metabolized to inosine by adenosine deaminase; however, our study failed to consistently track adenosine due to its transient nature and, as such, cannot confirm this [5,51].

Despite these observations, it is worth noting that the combination of allopurinol with azathioprine is a therapeutic option for patients who experience intolerance or side effects of azathioprine monotherapy, or for patients who cannot afford or do not have access to more recent biologic drugs [52]. This is attributed to the reduction in 6-methyl-mercaptopurine levels and the optimization of therapeutic 6-thiopurine production, which is enabled through inhibition of xanthine oxidase by allopurinol [53]. However, drugs of the thiopurine class are increasingly sparingly used in developed countries given their failure to demonstrate equivalent effectiveness when compared to modern biologics, which have faster onset therapeutic action and less adverse drug reactions [52,54]. While the risk of cancer with combination allopurinol and thiopurine still needs to be addressed with further studies [53], historical addition of allopurinol to thiopurine therapy has been shown to improve disease activity scores, promoting clinical remission, and, as such, still plays a valuable role in the treatment of IBD when thiopurine administration is required [54,55].

From our study, acute treatment with allopurinol appears associated with a marked loss in cellular energy (ATP) and simultaneous increases in phosphocreatine, which we postulate as indicative of disrupted purine nucleotide genesis and redirection of energy towards *de novo* purine biosynthesis. Conversely chronic treatment with allopurinol likely does not reflect the extent of metabolic perturbation at baseline as the tissue has adapted to any metabolic changes through compensatory mechanisms. However, it is apparent after insult that homeostasis is compromised. After epithelial insult, large quantities of energy and nucleotides for wound healing and proliferation are needed, but allopurinol inhibits the fundamental pathways needed to supply these. As such, the epithelium could not support itself via salvage, and, thus, likely had to utilize the costly *de novo* purine biosynthesis pathway due to accumulated Hpx, leading to further metabolic starvation. This observed spike in Hpx may be related to the reduction in AMPK expression we observed as well, given our immunofluorescence data and a recent study which found acute cellular stress-associated AMP to be rapidly converted to Hpx as a cellular mechanism to prevent premature and costly activation of the AMPK system [5]. As such, we argue for the capability of Hpx to act as a metabolite sink, where Hpx is generated from excess AMP but cannot be degraded. Since Hpx production is not allosterically regulated, AMP generated through cellular stress can be continually converted to Hpx, which is not salvaged back, and this action effectively buffers the activation of AMPK, despite the need for energy that excess AMP would generally signal. Intriguingly, this mechanism to buffer against premature AMPK activation, which would generally result in uric acid production, also suggests that chronic metabolic stress may also contribute to, or be a metabolic cause of, gout.

## 5. Conclusions

The present study highlights a concerning link between disrupted purine salvage by allopurinol and exacerbated colonic sensitivity to experimental colitis, thereby introducing concern regarding the GI safety profile of this widely used urate-lowering agent. The revelation that allopurinol introduces concerns regarding gut health encourages expanded investigation to delineate the precise mechanisms involved, be they related to a murine model only or translatable to relevant patient populations. It is reasonable that such an allopurinol influence translates to multiple organs. The consequential nature of these findings additionally highlights how our understanding of even older, widely used drugs is ever expanding and requires continued investigation to maximize therapeutic safety. Altogether, our work sheds light on a largely underexplored facet of gut health and demonstrates the capacity for allopurinol to perturb energy balance, predisposing the colonic epithelium to insult and slow remission from colitis.

## 6. Study Limitations

One limitation of the study was the use of dextran sodium sulfate, which induces acute colitis through epithelial damage and innate immune activation. This model is more relevant to ulcerative colitis and may not capture the full complexity of IBD. As such, care should be taken when interpreting these results, since allopurinol supplementation may trigger different responses in other IBD models. This work suggests the microbiota as a potentially significant component regarding the influence of allopurinol on intestinal homeostasis, and further study into how microbiota dysbiosis may contribute to impaired wound would be informative. Additionally, this study was intended to examine metabolism and, thus, contains little genetic or protein-based data to corroborate our findings regarding inhibition of XO, HGPRT, or PNP; as such, further study would be beneficial.

## Data Availability

The data presented in this study are available on request from the corresponding author, J. Scott Lee.

## Supplementary Materials

The following supporting information can be downloaded at: https://www.mdpi.com/article/10.3390/cells13050373/s1, Figure S1: Allopurinol inhibits proliferation in a dose-dependent manner in Caco-2C2bbe cells; Figure S2: Outcomes of in vitro allopurinol supplementation; Figure S3: Disease Activity Index (DAI) metrics during colitic induction and peak disease; Figure S4: Histological scoring during peak colitis.
