# Supplementary material for: Allopurinol Disrupts Purine Metabolism to Increase Damage in Experimental Colitis
Source: Cells. 2024 Feb 21;13(5):373. doi: 10.3390/cells13050373 (PMC10930830; doi:10.3390/cells13050373)
Supplement: Supplementary file 1 [file cells-13-00373-s001.zip › cells-2800910-supplementary.pdf]

Supplementary Materials

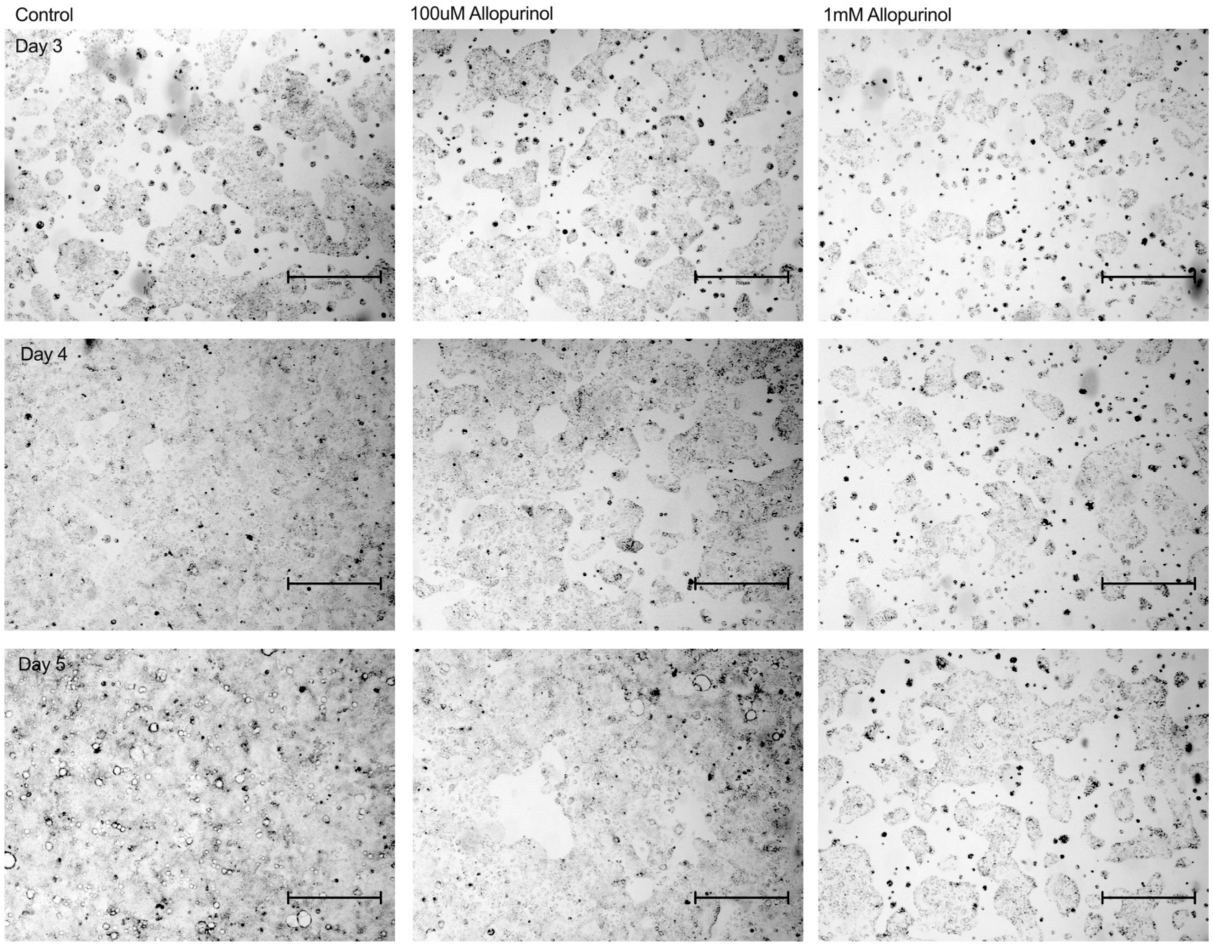

Figure S1: Allopurinol inhibits proliferation in a dose-dependent manner in Caco-2C2bbe cells. Scale bars, 750um.

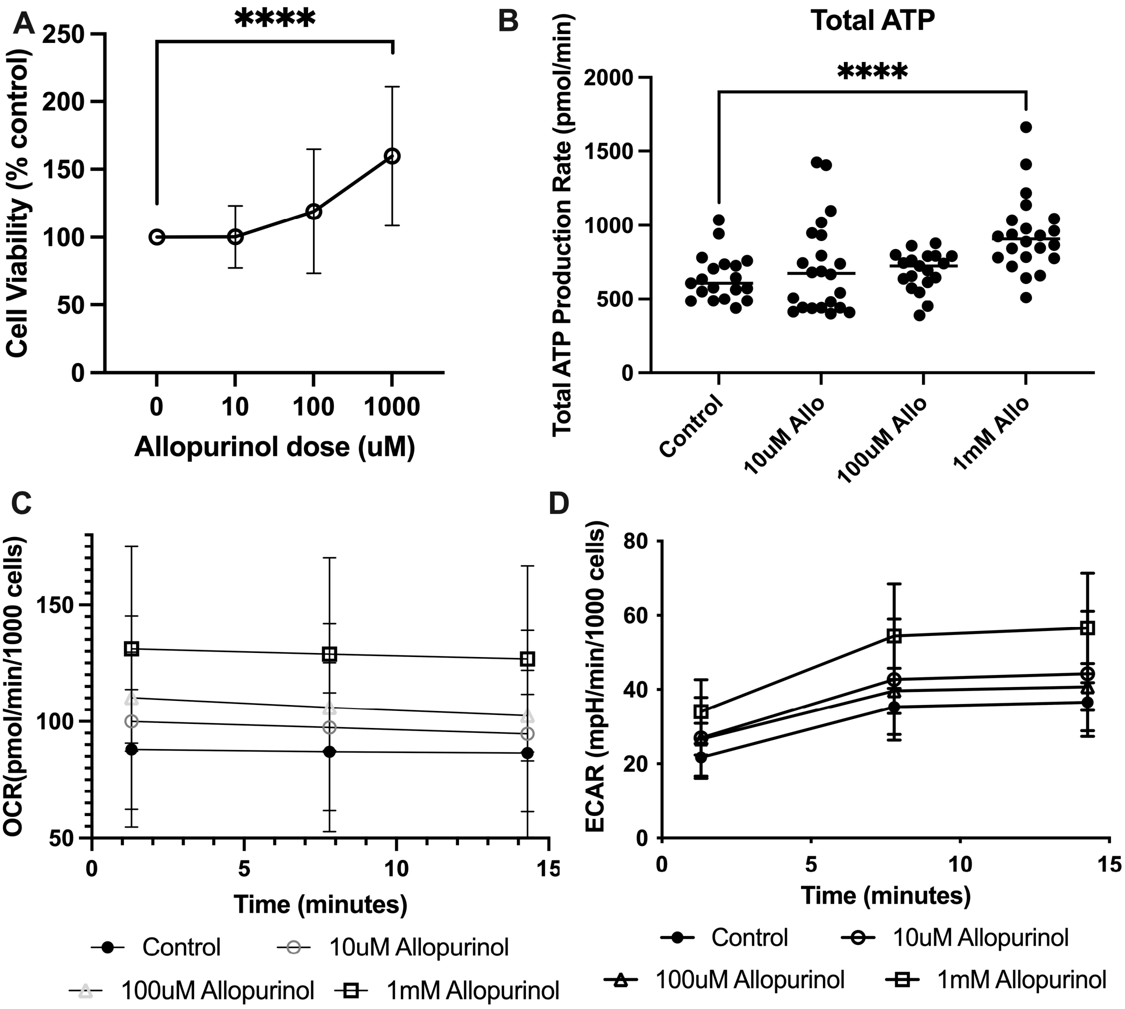

Figure S2: Outcomes of *in vitro* Allopurinol supplementation. (S2A) Cell viability of Caco-2C2bbe cells after 24 hours of allopurinol supplementation as measured by Cyquant MTT viability assay. (S2B) Total ATP production by allopurinol-treated T84 cells 24 hours following treatment, as measured by Seahorse XF Real-Time ATP Rate Assay. (S2C) Basal oxygen consumption rate of control and allopurinol-treated T84 cells as measured by Seahorse XF Real-Time ATP Rate Assay. (S2D) Extracellular acidification of control and allopurinol-treated T84 cells as measured by Seahorse XF Real-Time ATP Rate Assay.

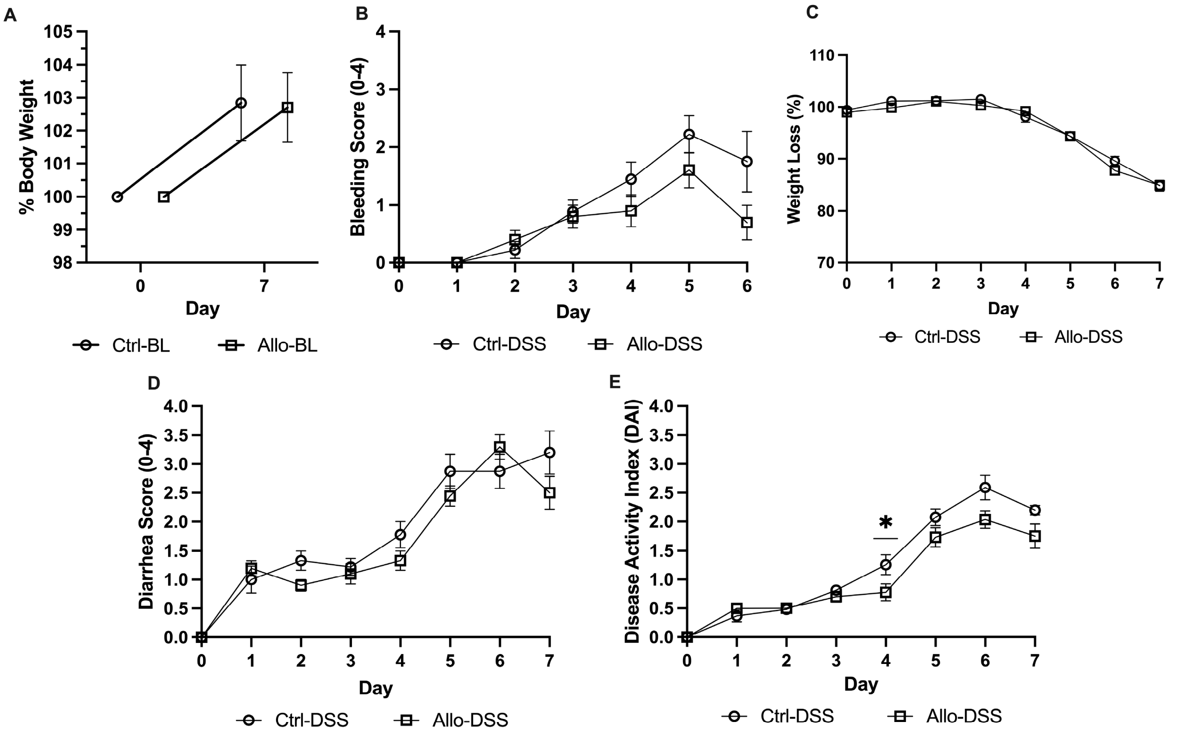

Figure S3: Disease Activity Index (DAI) metrics before and during colitic induction and peak disease. (S3A) Weight comparison of control and allopurinol-treated mice upon administration of allopurinol (Day 0), and after one week of allopurinol supplementation (Day 7). (S3B) Bleeding score of control and allopurinol-treated mice subjected to DSS colitis. (S3C) Percentage weight lost in control and allopurinol-treated mice due to DSS colitis. (S3D) Diarrhea score of control and allopurinol-treated mice due to DSS colitis. (S3E). Total disease activity index (DAI) scoring of colitis symptoms after induction of DSS-colitis.

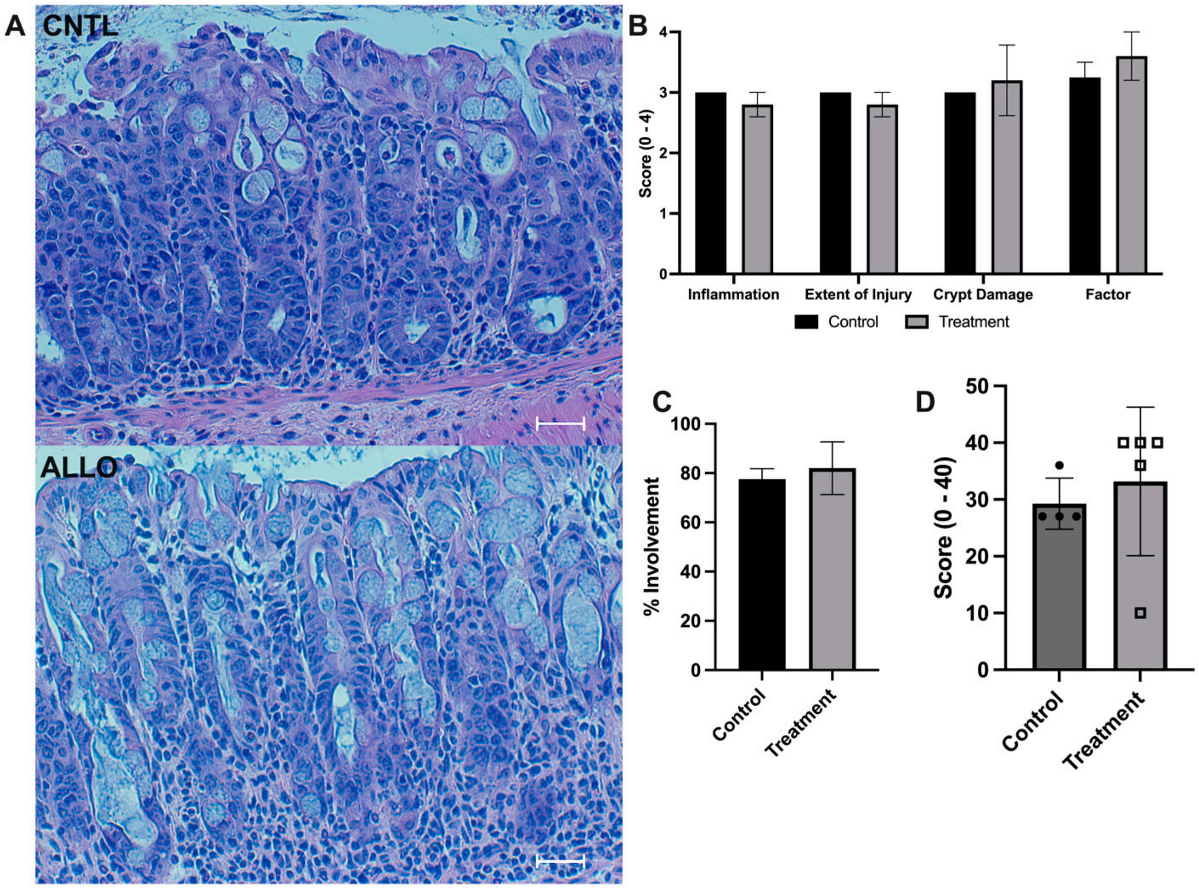

Figure S4: Histological scoring during peak colitis. (S4A) Representative H&E staining images of control (CNTL) and allopurinol-treated mice during peak colitis. Scale bars, 20µm. (S4B) Histological scoring of colitis symptoms. Three parameters measured were severity of inflammation (0-3: none, slight, moderate, severe), extent of injury (0-3: none, mucosal, mucosal and submucosal, transmural), and crypt damage (0-4: none, basal 1/3 damaged, basal 2/3 damaged, only surface epithelium intact, entire crypt and epithelium lost). The score of each parameter was multiplied by a factor reflecting the percentage of tissue involved (x1: 0-25%, x2: 26-50%, x3: 51-75%, x4: 76-100%). (S4C) Percentage of tissue affected in control (n = 4) and allopurinol-treated (n = 5) mice during peak colitis. (S4D) Total histological score of control and allopurinol-treated mice following colitis calculated by multiplying the score of each parameter by a factor reflecting the percentage of tissue involvement and all numbers were summed for a maximum possible score of 40. Data are presented as mean ± S.E. (error bars). *, $p < 0.05$.
